# Editorial: From Chronic Inflammation to Cancer: How Far Can Immunotherapy Go?

**DOI:** 10.3389/fphar.2021.838917

**Published:** 2022-01-12

**Authors:** Xuefeng Li, Lesheng Teng, Zhaogang Yang

**Affiliations:** ^1^ The Sixth Affiliated Hospital of Guangzhou Medical University, Qingyuan People’s Hospital, State Key Laboratory of Respiratory Disease, Sino-French Hoffmann Institute, School of Basic Medical Sciences, Guangzhou Medical University, Guangzhou, China; ^2^ School of Life Sciences, Jilin University, Changchun, China; ^3^ Department of Radiation Oncology, The University of Texas MD Anderson Cancer Center, Houston, TX, United States

**Keywords:** chronic inflammation, cancer, target therapy, immunotherapy, drug

Inflammation is divided into acute inflammation and chronic inflammation. Chronic inflammation has been proved to be one of the major culprits of tumor occurrence and development (Ameri et al., 2019). Once acute inflammation does not subside in time, it will turn into chronic inflammation, and then induce a variety of malignant tumors. Chronic inflammatory diseases, systemic chronic inflammation (obesity, depression, etc.,) and chronic inflammation caused by treatment can affect the immune system, thereby promoting the occurrence and development of tumors. In recent years, people have achieved many gratifying results, including in-depth research on the relationship between inflammation and tumors, the occurrence and regression mechanism of inflammation, and the role of chronic inflammation in tumorigenesis and development.

Here, we are committed to developing new strategies and treatments to activate the immune system of patients with chronic inflammation and cancer, in order to discover new drugs that can be used to combat chronic inflammation and induce cancer immune activation. In our research topic, Liu et al. described the role of IL-17A in Chronic Obstructive Pulmonary Disease (COPD), and discussed that IL-17A and its downstream regulators are potential therapeutic targets for COPD and subsequently COPD-derived lung cancer. Zhang et al. explored the role of FDX1 in the prognosis and metabolism of lung adenocarcinoma. They showed that FDX1 was closely related to glucose metabolism, fatty acid oxidation and amino acid metabolism. Another interesting study performed by Zeng et al. found chemokine ligand 14 (CXCL14) is involved in the proliferation and migration of ROS-induced colorectal cancer (CRC) cells, suggesting that aberrant ROS may promote colorectal cancer cell proliferation and migration through an oncogenic CXCL14 signaling pathway. Also, Wang et al., from another aspect, discussed the carnitine palmitoyltransferase system as a new target for anti-inflammatory and anti-cancer therapy, which may work as a promising anti-inflammatory/anti-tumor therapeutic strategy for numerous disorders. In tumor microenvironment field, Du et al. studied the tumor microenvironmental m6A as a prognostic biomarker associated with the hepatocellular carcinoma (HCC). And Wei et al. found that inhibition of BCL9 modulates the cellular landscape of tumor-associated macrophages in colorectal cancer.

Today, in cancer prevention and treatment, many clinical trials aiming at evaluating the efficacy of inflammatory regulators are underway. Though the single use of anti-inflammatory drugs has shown limited efficacy in cancer treatment, inflammation modulators can synergistically increase the anti-cancer drug’s efficacy in cancer therapies (chemotherapy, immunotherapy, etc.). For example, in some patients with advanced cancer, after conventional treatment, immunotherapy can still alleviate or even prevent the further development of disease (Ramagopalan et al., 2021). It is worth noting that the side effects suffered by patients during the remission period of several years are also lighter. Thus, there are still many key issues to be addressed on how to apply immunotherapy to regulate inflammation, or to improve the efficacy of cancer treatment or anti-cancer efficacy. For example: how to identify the most critical driving factors affecting the inflammatory response of cancer patients (Golder et al., 2021)? How to reduce or avoid side effects such as severe inflammation in the process of anti-cancer treatment (inflammatory storm induced by CAR-T treatment) (Hescot et al., 2018)? In addition, in the process of anti-tumor immunotherapy, we should also consider that different cancer patients will have different inflammatory responses. Personalized treatment strategies (precision medicine) for specific tumor-related inflammation will help improve anti-cancer efficacy. To answer these questions, our topic authors, through different ways and methods, in different tumors, have studied in depth some application cases of immunotherapy. Wu et al. explored T cell immunoreceptor with immunoglobulin and immunoreceptor tyrosine-based inhibitory motif domain (TIGIT), which is highly expressed in a subset of Treg cells, as a novel target for bladder cancer immunotherapy. Li et al. discussed the cGAS-STING pathway to explore the DNA immune recognition regulation in autoimmune disease and cancer. They summarized the current progress on cGAS-STING pathway modulators and laid the foundation for further investigating therapeutic development in autoimmune diseases and tumors. Wang et al. discussed the inhibitory effect of methylseleninic acid on esophageal squamous cell carcinoma through EGFR-IL-6 signaling axis. For metastatic cancer, Guo et al. used a xenograft model in zebra fish, and found lncRNA OR3A4 acts as the inflammatory cytokine to promote the proliferation and metastasis of ovarian cancer through KLF6 pathway.

In summary, the collection of aforementioned articles in this research topic provide either overview of novel targets in immunotherapy, or new fundamental findings and summaries related to chronic inflammation and cancer. Additional researches, for example, drug delivery systems and synthetic antibody engineering, are needed to gain further insights as we move toward to improve the safety and efficacy of novel immunotherapy applications in chronic inflammation and cancer.
